# Hemorrhagic Descemet Membrane Detachment during Ab Interno Canaloplasty

**DOI:** 10.1155/2019/3653954

**Published:** 2019-04-21

**Authors:** Juan Carlos Izquierdo Villavicencio, Josefina A. Mejías Smith, Laura A. Cañola Ramírez, Natalia Agudelo Arbelaez, Bárbara Rubio Lastra

**Affiliations:** ^1^Glaucoma Research Department, Instituto de Ojos Oftalmosalud, Lima, Peru; ^2^Glaucoma Department, Instituto de Ojos Oftalmosalud, Lima, Peru

## Abstract

**Purpose:**

To describe a Descemet membrane detachment in peripheral cornea after canaloplasty with ab interno approach in glaucoma.

**Case Report:**

A 60-year-old male with uncontrolled primary open-angle glaucoma (POAG) underwent ab interno canaloplasty in the left eye. The previous corrected visual acuity was 20/400 and intraocular pressure 26 mmHg with maximum medical therapy. There was evidence of minor intrastromal bleeding and limited Descemet membrane detachment during the introduction of intracanalicular viscoelastic. Speculate that the Descemet detachment occurred owing to the excessive pressure while injecting the viscoelastic. A conservative management was decided due to the size of the detachment outside the visual axis. On the first postsurgical day, the slit lamp biomicroscopy confirmed that the paralimbal extension of the pre-Descemet hemorrhage was 3mm and the radial extension was 2mm. Moreover the initial thickness of the pre-Descemet hemorrhage measurement with anterior segment OCT was 0.6mm. The follow-up was done weekly. At 3 months postoperatively, cornea recovered its transparency and morphology and intraocular pressure was 18mmHg with maximum medical therapy.

**Conclusion:**

Descemet membrane detachment by viscoelastic with partial intrastromal hematoma is a rare complication of the ab interno canaloplasty, which can be managed conservatively if it has not compromised the visual axis and has a limited extension.

## 1. Introduction

Ab interno canaloplasty is a nonpenetrating glaucoma surgery which increases the drainage of aqueous humor from the anterior chamber through the trabecular meshwork to the Schlemm canal [1]; it is safe and effective and it may be conducted with other surgeries [2, 3]. Its purpose is to renew entire drainage system in a more physiological way which differs from the conventional glaucoma surgeries [4, 5].

The ab interno canaloplasty was discussed because according to Lewis et al., some patients undergoing ab external canaloplasty could only have viscodilation, and during the monitoring process to these patients after 3 years, it was evidenced that the results obtained were successful despite the fact that the technique was not complete [4]. This new approach was described when the Schlemm canal is dilated through viscoelastic and the trabecular meshwork is extended, thus creating microfractures in 360° circumferential direction to the canal [2, 6].

This article is the first report related to hemorrhagic Descemet membrane detachment due to viscoelastic in ab interno approach and we opted for observative management due to the extent of the injury, evidencing favorable results for the patient during 3-month monitoring process.

## 2. Case Report

A 60-year-old man with advanced bilateral open-angle glaucoma for 3 years, not controlled with maximum medical therapy in the left eye, underwent phacoemulsification in the left eye in 2014. The medical records did not register coagulation disorders and the patient denied to have taken antiplatelet medicine or anticoagulants.

Before the surgery, the uncorrected visual acuity (UCVA) was 2.5 and 1.3 LogMAR in the right and left eye, respectively. The intraocular pressure was 12mmHg in the right eye and 26mmHg in the left eye with maximum medical therapy. On Humphrey Field Analyzer (HFA) 24-2 automated perimetry mean deviation (MD) was -16.92 in the left eye, pachymetry was 517 microns in the right eye and 492 microns in the left eye, and the number of central endothelial cells was 2127 cells/mm2 in the right eye and 1312 cells/mm^2^ in the left eye.

Ab interno canaloplasty was performed routinely in the left eye by a glaucoma specialist. The temporary corneal incision was made at hour 9 and another lateral incision was made at hour 2 to introduce the iTrack catheter (iTrack-250A; iScience Interventional, Menlo Park, CA, USA). Sodium hyaluronate was injected (Healon GV; Abbott Medical Optics, Santa Ana, CA, USA) in the anterior chamber. Gonioscopy was used (AVG; Surgical Gonio Lens, Volk Alcon, Mentor, OH, USA) for goniotomy with Kahook dual blade (KDB; New World Medical, Rancho Cucamonga, CA, USA), and by using tying forceps (Intraocular Tying Forceps, 23G 4-1891, Rumex, USA) the catheter was pushed circumferentially through 360°, by applying two viscoelastic clicks per hour when removing it.

**Figure 1:**
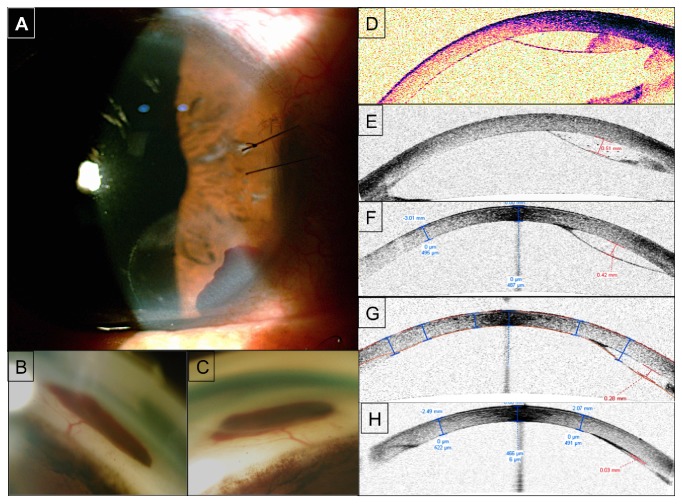
(A) First day after the surgery. Picture of the slit lamp showing a Descemet membrane detachment with partial intrastromal hemorrhage. (B) Gonioscopy evidencing Descemet detachment with intrastromal hemorrhage, 1st day after the surgery. (C) Gonioscopy evidencing Descemet detachment with intrastromal hemorrhage 1st month after the surgery with a reduction of the extent of the injury. (D) OCT of anterior segment (Visante, Model 1000, Carl Zeiss Meditec*, Dublin CA, USA), showing *intrastromal hemorrhage. (E) OCT Anterior Section, second week after the surgery (4 January 2018). (F) OCT Anterior Section, sixth week after the surgery (2 February 2018). (G) OCT Anterior Section, tenth week after the surgery (1 March 2018). (H) OCT Anterior Section, twelfth week after the surgery (19 March 2018).

The procedure was performed correctly; however, during the viscodilation of the Schlemm canal with sodium hyaluronate, a small hemorrhage was observed with viscoelastic related to the Descemet membrane detachment. The paralumbar extension was 3.0 mm and radial extension was 2.0 mm between hours 4 and6 in peripheral inferonasal quadrant. At first we opted for observation because the injury did not compromise the visual axis and the size of the detachment was not large enough to indicate a surgical procedure immediately (Figure 1(A)).

A serial control with anterior segment OCT (Visante, Model 1000, Carl Zeiss Meditec, Dublin CA, USA) has been done to follow the thickness; the initial thickness of intrastromal hemorrhage was 0.6mm, and at the first week it was 0.51mm, at the first month 0.42mm, and at the second month 0.28 mm and third month 0.03 mm. The examination evidenced presence of blood in the peripheral inferonasal quadrant of the pre-Descemet area at hours 4-6 (Figures 1(D), 1(E), 1(F), 1(G), and 1(H)).

Considering the extension of the detachment, a conservative management was decided, monitoring the progressive reabsorption of the hemorrhage and viscoelastic, which progressively occurred (Figures 1(B) and 1(C)).

The intraocular pressure levels remained lower than 21mmHg in the early postoperative period with glaucoma medications; 3 months after the surgery the intraocular pressure was 18mmHg with 3 antiglaucoma medications. The uncorrected visual acuity (UCVA) showed a significant improvement from 1.30 in the preoperative period to 0.8 LogMAR in the left eye after 3 months of monitoring (Table 1). Final BCVA was 0.6 LogMAR.

The number of central endothelial cells registered a small decrease from 2,127 to 1,809 cells/mm^2^ in the third month with a 14.9% loss. Final pachymetry was 553 microns in the left eye, showing an increase of corneal thickness of 12.39% in the third month.

According to Hodapp classification, the visual field defect 10-2, stimulus III, and white-on-white of the left eye indicated a stable advanced stage glaucoma with a MD of -31.4.

Three months after the surgery, the Descemet membrane detachment with intrastromal hematoma completely recovered; the membrane was reattached correctly (Figure 1(H)) and remained that way during the monitoring with the transparent cornea along with no visual consequences.

## 3. Discussion

The hemorrhagic Descemet membrane detachment with viscoelastic is a rare complication reported in the canaloplasty with external approach during the catheterization of Schlemm canal when injecting high-molecular-weight viscoelastic substances [5, 7]. Yalvac et al. reported one Descemet detachment followed by hemorrhage from the episcleral vessels or by blood reflux from the Schlemm canal [8]. This complication may cause a significant reduction of visual acuity if compromising the visual axis, according to the size and location of detachment.

The incidence related to the Descemet membrane detachment has been reported up to 7.4%, and in most cases, it is related to excessive viscoelastic during its injection [9]. Alobeidan et al. reported an incidence of 6.7% in most external phacocanaloplasty procedure [1].

The Descemet detachment may occur during the viscoelastic abundant injection or when the microcatheter does not move to the exit continuously; thus viscoelastic may enter to the cornea at Schwalbe's line and blood may flow back from collector canals [10]. To reduce the risk of overexpanding the Schlemm canal during canaloplasty, two clicks must be injected per hour, always considering that there is a delay between the click and the viscoelastic flow in the catheter. It is important to hear the injector click [2]. Most of the reported Descemet membrane detachments [11] with intrastromal hemorrhage after ab externo canaloplasty were located in the inferonasal quadrant [1, 2, 5].

Jaramillo et al. [12] suggested performing surgery in this complication in case the hemorrhage is over 3mm and close to the visual axis, so based on previous experience and reported cases, we decided a conservative management despite the fact that there are reports where the spontaneous resolution may take from 6 months to 2.5 years [10, 13].

Lewis et al. presented a 2-year follow-up of patients after ab externo canaloplasty, finding a 30% decrease in IOP [12]. In our patient the complication did not alter the expected outcome of the procedure, because after three months about 30% IOP decrease was recorded with the same number of glaucoma medications. Interestingly according to a study no significant IOP lowering difference was found after ab externo and ab interno canaloplasty in a series of twelve patients who underwent ab interno in one eye and ab externo canaloplasty in the other eye [14].

The ab interno canaloplasty is effective and less traumatic than ab externo canaloplasty. It requires no two scleral flap dissections to expose Schlemm canal and Descemet window and no placement of tensioning suture, as in ab externo procedure, and allows the expansion of both Schlemm canal and collector canals. However, also in ab interno canaloplasty, maneuvers that could lead to the Descemet membrane detachment should be avoided [3]. Quite recently in a consecutive series of 20 ab interno canaloplasties one limited descemetolysis near the limbus by the viscoelastic during the dilation of Schlemm canal was reported [15]. In our case observation proved to be a wise decision regarding the hemorrhagic Descemet membrane detachment following ab interno canaloplasty. The expectant management is a feasible option when the extension and thickness of intrastromal hemorrhage are limited and do not involve the visual axis.

The Descemet detachment with intrastromal hemorrhage is a rare complication after ab externo canaloplasty. The nonconservative management options include partial thickness paracentesis [5], intracameral SF6 [1], and intracorneal blood with balanced saline solution following deep corneal incision and air bubble injection of the shallow anterior chamber [13].

## Figures and Tables

**Table 1:** Monitoring of Visual acuity, intraocular pressure and number of medications.

Periods	UCVA(LogMAR)	IOP (mmHg)	Medications
Preoperative	1.30	26	3
Day 1	2.30	40	4
Week 1	2.10	14	3
Month 1	0.80	14	3
Month 2	0.80	8	2
Month 3	0.80	18	3
